# Latent profiles and influencing factors of missed nursing care among ICU nurses

**DOI:** 10.1186/s12912-026-05308-1

**Published:** 2026-08-28

**Authors:** Xiangyue Liu, Xue Deng, Bo He, Wenling Tian, Lin Cui, Dongfa Liao, Dongwen Li, Jing Cui, Ye Bi, Qian Tan

**Affiliations:** 1 College of Nursing, Chengdu Medical College, Chengdu, Sichuan 610000 China; 2 Orthopaedics Department, The General Hospital of the Western Theater Command of the People’s Liberation Army, Chengdu, Sichuan 610000 China; 3 College of Nursing, North Sichuan Medical College, Nanchong, Sichuan 637000 China; 4 Nursing Department, The General Hospital of the Western Theater Command of the People’s Liberation Army, Chengdu, Sichuan 610000 China; 5 Stomatology Department, The General Hospital of the Western Theater Command of the People’s Liberation Army, Chengdu, Sichuan 610000 China; 6 Orthopaedics Department, The 920th Hospital of the Joint Logistic Support Force of the Chinese People’s Liberation Army, Kunming, Yunnan, 650000 China; 7 Outpatient Department, The 920th Hospital of the Joint Logistic Support Force of the Chinese People’s Liberation Army, Kunming, Yunnan, 650000 China

**Keywords:** Missed nursing care, Intensive care units, Nurses, Latent profile analysis, Moral sensitivity, Perceived organizational support

## Abstract

**Background:**

Missed nursing care (MNC) is common in intensive care units (ICUs) and seriously threatens patient safety. Most existing studies treat ICU nurses as a homogeneous group, overlooking within-group heterogeneity. Based on the Job Demands-Resources model, this study aimed to identify latent profiles of missed nursing care among ICU nurses and examine their influencing factors.

**Methods:**

A cross-sectional study was conducted from November 30, 2025, to March 31, 2026. A convenience sample of 332 ICU nurses was recruited from five tertiary Grade A hospitals in Sichuan, China. Data were collected using the Missed Nursing Care Scale, Moral Sensitivity Questionnaire, and Perceived Organizational Support Questionnaire. Latent profile analysis, correlation analysis, hierarchical regression, and multinomial logistic regression were used for analysis.

**Results:**

The overall score of missed nursing care was 67.54 ± 21.33. Three subgroups were identified: low (56.63%), moderate (28.31%), and high (15.06%) missed nursing care. The high group had the highest missed care scores and the lowest perceived organizational support, while the low group had the highest moral sensitivity and the highest proportion of ICU specialist nurses. The pattern of missed nursing care differed across profiles: the low group mainly reported omissions in supportive nursing activities, the moderate group showed additional deficits in care planning, and the high group demonstrated omissions in core clinical care activities, including sedation and analgesia management. The total moral sensitivity score was negatively associated with missed nursing care overall, and this association was significant only in the low MNC subgroup. Lower perceived organizational support was associated with membership in the high MNC group relative to the moderate group. Hierarchical regression identified monthly overtime, dissatisfaction with human resource allocation and communication climate, ICU specialist nurse qualification, moral sensitivity, and perceived organizational support as factors associated with missed nursing care.

**Conclusions:**

Missed nursing care among ICU nurses shows clear heterogeneity and can be divided into three subgroups. The associations of moral sensitivity and organizational support might be context-dependent. Stratified interventions tailored to subgroup characteristics may help address missed nursing care, though their effectiveness requires testing in future studies.

**Trial registration:**

Not applicable (this study is an observational study, not a randomized controlled trial).

**Clinical trial number:**

Not applicable.

**Supplementary Information:**

The online version contains supplementary material available at https://doi.org/10.1186/s12912-026-05308-1.

## Introduction

The Intensive Care Unit (ICU) provides specialized care for critically ill patients. ICUs are characterized by complex and unstable conditions, frequent invasive procedures, heavy workloads, and emergency resuscitations. Common challenges include insufficient staffing, nurse burnout, a relatively young nursing workforce, and limited specialized competence [1]. These issues seriously compromise nursing quality and patient safety and have attracted considerable attention. International nursing organizations, including the International Council of Nurses, have repeatedly called for optimizing nurses’ work environments and ensuring adequate nursing staffing to improve patient safety and care quality [2]. In this context, missed nursing care (MNC) has gradually gained attention. MNC refers to the partial or complete failure or delay in delivering necessary nursing interventions for patients due to various reasons [3, 4]. MNC not only endangers patient safety and increases the risk of adverse outcomes, such as pressure injuries, healthcare-associated infections, and medication errors [5], but also reduces nurses’ job satisfaction and exacerbates burnout and turnover intention. Compared with general ward nurses, ICU nurses face higher patient acuity, more frequent emergencies, and greater task intensity [6]. Moreover, most ICU patients are unconscious and equipped with multiple invasive tubes [7], requiring higher standards of basic and specialized nursing; therefore, the consequences of missed nursing care can be particularly severe [8]. Studies have shown that 73.4% to 86.0% of ICU nurses report experiencing missed nursing care [9, 10], and a Chinese survey indicated that 82.14% of ICU nurses had encountered at least one type of missed nursing care [11]. The RN4CAST multi-country study showed that comforting/talking with patients and developing or updating care plans were among the most frequently omitted nursing activities in European hospitals [12]. Evidently, missed nursing care has become a major threat to ICU nursing quality and patient safety.

Most current studies on ICU missed nursing care employ variable-centered approaches. These studies treat ICU nurses as a homogeneous group and only report overall levels of missed nursing care, overlooking potential within-group heterogeneity. Even among nurses who experience missed nursing care, notable differences exist in the type, frequency, and severity of omitted items. However, the links between these differences and individual nurse characteristics remain unclear. This limits managers’ ability to design personalized interventions. The Job Demands-Resources (JD-R) model provides a theoretical framework for this study. The model [13–15] proposes that job demands consume employees’ energy, whereas job resources buffer their negative effects and promote positive work outcomes. European research has also shown that missed nursing care serves as a mediating factor between nurse staffing and perceived quality of care as well as burnout risk [16], indicating that missed nursing care is not merely a resource issue but also involves complex relationships with work environment and nurse outcomes. In this study, job demands were operationalized as average number of patients per shift and average monthly overtime hours, whereas job resources included ICU specialist nurse qualification, satisfaction with human and material resource allocation and communication climate, moral sensitivity, and perceived organizational support. ICU specialist nurse qualification and moral sensitivity were conceptualized as individual resources, whereas satisfaction with resource allocation and communication climate and perceived organizational support were considered organizational resources. Accordingly, this study considered moral sensitivity as an internal resource within the JD-R framework. Moral sensitivity reflects nurses’ ability to recognize ethical conflicts and understand professional responsibilities. Perceived organizational support was used as an organizational resource. It reflects the extent to which nurses feel valued and supported by their institution. Both factors may help nurses prioritize patient care under high job demands and thus address missed nursing care.

However, existing studies have not clearly demonstrated whether the associations of moral sensitivity and perceived organizational support with missed nursing care vary across subgroups characterized by different levels of missed nursing care. In addition, these studies have not identified the latent profile characteristics of missed nursing care among ICU nurses. Therefore, this study used latent profile analysis (LPA) to classify ICU nurses. We explored the characteristics and distribution patterns of different subgroups of missed nursing care and identified profiles with higher levels of missed nursing care. We further analyzed the heterogeneous associations of moral sensitivity and perceived organizational support across these subgroups. The findings provide empirical evidence for developing stratified and targeted interventions. Such interventions may help reduce missed nursing care and could contribute to improving ICU nursing quality and patient safety, though this requires confirmation in future intervention studies.

## Participants and methods

### Setting

This research was conducted in the intensive care units (ICUs) of five tertiary Grade A hospitals in Sichuan Province, Southwest China. All hospitals are comprehensive medical institutions providing critical care services.

### Participants

A convenience sampling method was adopted. From November 30, 2025 to March 31, 2026, ICU nurses from 5 tertiary Grade A hospitals were recruited as study participants. For multiple regression, Kendall’s rule of thumb recommends a sample size of 5 to 10 times the number of independent variables [17]. To provide a conservative estimation, the maximum number of variables assessed in this study was considered, including demographic and work-related characteristics, individual missed nursing care items, perceived causes dimensions, moral sensitivity dimensions, and perceived organizational support. Therefore, 58 variables were considered for sample size estimation. Based on the minimum recommended ratio of 5 times the number of variables, the initial estimated sample size was 290 cases. After accounting for a 10% invalid questionnaire rate, the target sample size was set at approximately 323 cases. For LPA, a sample size of at least 300 is recommended to ensure robust classifications [18]. The sample size was determined based on the requirements of multinomial logistic regression and LPA. The final valid sample in this study was 332 cases, meeting the requirements for both analytical approaches.

**Inclusion criteria**: (1) Registered nurses with a valid nursing license currently working in the ICU of the included hospitals; (2) At least 1 year of clinical working experience in the ICU; (3) Voluntary participation in this study. **Exclusion criteria**: (1) ICU nurses engaged in non-clinical nursing work; (2) Intern nurses, visiting nurses, and standardized training nurses.

### Measures

#### General information questionnaire

A general information questionnaire was developed specifically for this study based on a systematic literature review and study aims, comprising 13 variables: gender, age, marital status, education level, professional title, ICU type, years of ICU work experience, average number of patients per shift, average monthly overtime hours, ICU specialist nurse qualification, satisfaction with departmental human resources, satisfaction with material resources, and satisfaction with departmental communication climate. A pilot test was conducted among 30 ICU nurses to confirm its readability and comprehensibility. As this questionnaire collected only demographic and work-related information, formal reliability and validity testing was not performed; however, the items were derived from the literature and reviewed by the research team, and no ambiguities were identified in the pilot test.

#### Intensive Care Unit Missed Nursing Care Scale

The Intensive Care Unit Missed Nursing Care Scale was developed by Liu Yu’an et al. [1]. (China) in 2024 to assess the status and causes of missed nursing care among ICU nurses. Part A of the scale covers missed nursing care items, consisting of individual nursing activities without predefined subdimensions. It includes three domains: disease monitoring and evaluation (18 items), basic nursing care (13 items), and specialized nursing care (6 items), totaling 37 items. A 5-point Likert scale was used (1–5 points), with total scores ranging from 37 to 185; higher scores indicate a higher level of missed nursing care. Part B includes 25 items measuring perceived causes of missed nursing care, divided into 5 dimensions, rated on a 4-point Likert scale (1 = not a cause, 2 = minor cause, 3 = moderate cause, 4 = major cause). Higher scores represent more influential causes of missed nursing care. The Cronbach’s α coefficients for Part A and Part B were 0.963 and 0.946, respectively, and those for the subdimensions of Part B ranged from 0.708 to 0.904, indicating good reliability and validity for this study.

#### Moral Sensitivity Questionnaire

The Moral Sensitivity Questionnaire was originally developed by Lützén et al. [19] in 2001 and revised in 2006 for healthcare professionals to assess moral sensitivity among nurses with different characteristics; the overall Cronbach’s α coefficient was 0.780. Huang et al. [20] introduced the Chinese version in 2016 for Chinese nurses, with an overall Cronbach’s α coefficient of 0.820. The scale comprises two dimensions: moral responsibility and strength (5 items) and moral burden (4 items), totaling 9 items. A 6-point Likert scale was used (1 = strongly disagree to 6 = strongly agree), with total scores ranging from 9 to 54. Higher scores reflect stronger moral sensitivity. The scale has good reliability and validity and was adopted in this study. Moral responsibility and strength reflect the positive dimension of moral sensitivity, including the courage to act on behalf of patients and the sense of responsibility for providing good care. Moral burden reflects the negative dimension of moral sensitivity, referring to the emotional distress and cognitive awareness that arise when nurses recognize ethical challenges and feel unable to fully address them. Unlike economic, psychological, or social burdens, moral burden is specifically caused by problems or situations that involve moral values [19].

#### Nurse Perceived Organizational Support Questionnaire

The Nurse Perceived Organizational Support Questionnaire was developed by Wang Haocen et al. [21] in 2014. It is unidimensional with 15 items, rated on a 5-point Likert scale (1 = strongly disagree to 5 = strongly agree). Higher scores indicate higher perceived organizational support. Exploratory factor analysis showed a cumulative variance contribution rate of 60.825%, the content validity index was 0.940, Cronbach’s α coefficient was 0.953, split-half reliability was 0.909, and test-retest reliability was 0.812, confirming good reliability and validity for this study. All the above questionnaires have been authorized by the researchers. The survey was administered using paper questionnaires. Each participant received a questionnaire in a sealed envelope from trained research assistants, completed it anonymously, and returned it in the sealed envelope on site.

### Statistical analysis

Statistical analyses were performed using SAS 9.4 and Mplus 8.3. Two-sided tests were used with a significance level of α = 0.05. Categorical data were described by frequency and constituent ratio. After normality testing via Q-Q plots confirmed that the data were approximately normally distributed, measurement data were expressed as mean ± standard deviation (mean ± SD). For univariate analysis, independent-samples t-test was used for comparisons between two groups, one-way analysis of variance (ANOVA) for multiple groups, and chi-square test for categorical variables. When the overall ANOVA was statistically significant, pairwise comparisons were performed using the least significant difference (LSD) test.

LPA was conducted using Mplus 8.3, with the mean scores of the three content domains of Part A of the ICU Missed Nursing Care Scale as indicators. Models with 1 to 5 latent classes were fitted sequentially. Model fit was evaluated using the Akaike Information Criterion (AIC), Bayesian Information Criterion (BIC), adjusted Bayesian Information Criterion (aBIC), Entropy, Lo-Mendell-Rubin likelihood ratio test (LMR), and Bootstrap-based likelihood ratio test (BLRT). Lower AIC, BIC, and aBIC values, Entropy > 0.8, and significant LMR and BLRT tests (*P* < 0.05) indicated better model fit.

Pearson correlation analysis was used to examine the relationships between missed nursing care, moral sensitivity, and perceived organizational support.

For hierarchical multiple linear regression, the total missed nursing care score was used as the dependent variable. Predictors were entered using a theory-driven hierarchical enter approach based on the JD-R framework. Block 1 included demographic and general work-related characteristics; Block 2 additionally included job-demand variables; and Block 3 additionally included job-resource variables. All predictors within each block were entered simultaneously, and no variables were automatically selected or removed according to statistical significance.

Categorical predictors were dummy-coded, with selected sparse categories combined before multivariable modeling to improve estimate stability. Age was categorized as ≤ 25, 26–30, 31–35, and ≥ 36 years, with ≥ 36 years as the reference category. Education was categorized as junior college or below versus bachelor’s degree or above, with bachelor’s degree or above as the reference category. Professional title was categorized as nurse, senior nurse, and nurse-in-charge or above, with nurse-in-charge or above as the reference category. ICU type was categorized as medical ICU, surgical ICU, and general/emergency ICU, with general/emergency ICU as the reference category. Years of ICU experience were retained as 1–4, 5–9, and ≥ 10 years, with 1–4 years as the reference category. Average patient load per shift was retained as 1–2, 3–4, and ≥ 5 patients, with 1–2 patients as the reference category. Average monthly overtime was retained as no overtime, 1–5, 6–10, 11–15, and > 15 h, with no overtime as the reference category.

For descriptive and univariate analyses, the three satisfaction variables were retained in their original four categories (very dissatisfied, dissatisfied, satisfied, and very satisfied) to preserve the full distribution of responses. For multivariable regression analyses, satisfaction with human resources, material resources, and communication climate was dichotomized as dissatisfied (very dissatisfied/dissatisfied) versus satisfied (satisfied/very satisfied), with satisfied as the reference category. The same dichotomous definition was used in both the hierarchical linear regression and multinomial logistic regression. Gender, marital status, and ICU specialist nurse qualification were binary variables, with male, unmarried, and non-specialist nurse, respectively, serving as the reference categories. Moral responsibility and strength, moral burden, and perceived organizational support were entered as continuous variables.

For the hierarchical multiple linear regression, multicollinearity was assessed using tolerance and variance inflation factor (VIF) values, with VIF < 5 indicating no substantial multicollinearity. Linear regression assumptions were further evaluated using standardized residuals, normal probability plots, standardized residual-versus-predicted-value plots, Cook’s distance, leverage values, and the Durbin-Watson statistic.

For multinomial logistic regression, latent profile membership (low, moderate, and high missed nursing care) was used as the dependent variable. Demographic and work-related variables showing statistically significant differences across the three profiles in the univariate comparisons were entered into the model, together with the two dimensions of moral sensitivity (moral responsibility and strength and moral burden) and perceived organizational support, to examine factors associated with latent profile membership. The moderate missed nursing care group was set as the reference category. Categorical predictors were dummy-coded using the reference categories specified above, and the three satisfaction variables were entered using the same dichotomous coding as in the hierarchical linear regression. Odds ratios (ORs) and 95% confidence intervals (CIs) were reported. Overall model significance was assessed using the likelihood-ratio test, and Cox-Snell, Nagelkerke, and McFadden pseudo-R² measures were reported. Multicollinearity was assessed using variance inflation factors (VIFs), with values < 5 indicating no substantial multicollinearity.

### Ethical considerations

This study was approved by the Biomedical Ethics Committee of Chengdu Medical College (approval number: CMCIR2025NO.040).

An informed consent form was attached to the front page of the questionnaire, clarifying the research purpose, data usage, and confidentiality rules to participants. Data were collected by trained research assistants using paper questionnaires enclosed in sealed envelopes. Informed consent was obtained when participants signed and dated the form after full reading. Participants had the right to voluntarily decide whether to participate, refuse to provide information, or withdraw at any time without any adverse consequences.

Privacy of participants was strictly protected during the research process. All data were used only for this study, stored in locked cabinets and password-protected electronic files accessible only to the research team, and destroyed after study completion. No individual identifying information will be disclosed in published results.

## Results

### Participant characteristics

A total of 369 questionnaires were distributed, and 332 valid questionnaires were collected, with an effective response rate of 89.97%. Questionnaires with incomplete data or invalid responses were excluded, and no missing data were present in the final analysis dataset. The demographic and work-related characteristics of the participants are summarized in Table 1.

**Table 1:** Demographic characteristics and univariate analysis of missed nursing care among ICU nurses (*N* = 332)

Demographic variables	Frequency	Composition ratio (%)	Mean ± Standard deviation	F/t	*P* value
**Gender**				0.149*	0.881
Male	68	20.5	67.19 ± 20.05		
Female	264	79.5	67.63 ± 21.69		
**Age (years)**				5.674**	0.001
≤ 25	50	15.1	68.14 ± 14.46^a^		
26–30	101	30.4	61.53 ± 20.94^a^		
31–35	121	36.4	66.64 ± 20.80^a^		
36–40	54	16.3	80.30 ± 23.84^b^		
≥ 41	6	1.8	66.67 ± 19.81^ab^		
**Marital status**				1.693*	0.091
Married	174	52.4	69.40 ± 23.72		
Unmarried	158	47.6	65.49 ± 18.22		
**Education level**				1.456**	0.279
Technical secondary school	5	1.5	71.00 ± 24.99		
Junior college	64	19.3	63.27 ± 17.04		
Bachelor	257	77.4	68.41 ± 22.26		
Postgraduate	6	1.8	72.67 ± 17.22		
**Professional title**				5.944**	0.001
Nurse	48	14.5	68.08 ± 11.03^ab^		
Senior nurse	172	51.8	63.52 ± 21.17^a^		
Nurse-in-charge	110	33.1	73.88 ± 23.53^b^		
Associate chief nurse	2	0.6	51.00 ± 12.73^ab^		
**ICU specialist nurse**				7.888*	<0.001
Yes	147	44.3	58.32 ± 15.82		
No	185	55.7	74.86 ± 22.33		
**Type of ICU**				4.515**	0.006
Medical ICU	62	18.7	61.53 ± 18.93^a^		
Surgical ICU	85	25.6	72.59 ± 23.61^b^		
General ICU	171	51.5	66.59 ± 20.81^ab^		
Emergency ICU	14	4.2	75.00 ± 15.11^ab^		
**Working years in ICU (years)**				1.904**	0.152
1–4	155	46.7	65.27 ± 20.63		
5–9	80	24.1	70.80 ± 21.82		
≥ 10	97	29.2	68.46 ± 21.83		
**Average number of patients per shift (persons)**				6.651**	0.002
1–2	82	24.7	60.59 ± 19.20^b^		
3–4	220	66.3	69.91 ± 21.66^a^		
≥ 5	30	9.0	69.13 ± 21.00^ab^		
**Average monthly overtime hours (hours)**				9.942**	<0.001
No overtime	54	16.3	58.80 ± 14.67^a^		
1–5	158	47.6	63.95 ± 20.74^ac^		
6–10	58	17.5	76.28 ± 21.17^b^		
11–15	30	9.0	76.07 ± 23.66^bc^		
>15	32	9.6	76.16 ± 21.83^bc^		
**Satisfaction with departmental human resource allocation**				32.425**	<0.001
Very satisfied	10	3.0	50.70 ± 6.65^a^		
Satisfied	119	35.8	59.18 ± 17.42^b^		
Dissatisfied	177	53.3	71.39 ± 21.51^c^		
Very dissatisfied	26	7.8	86.04 ± 20.33^d^		
**Satisfaction with departmental material resource allocation**				25.133**	<0.001
Very satisfied	26	7.8	52.73 ± 8.24^a^		
Satisfied	102	30.7	62.94 ± 21.84^b^		
Dissatisfied	183	55.1	70.93 ± 20.62^c^		
Very dissatisfied	21	6.3	78.57 ± 23.27^bc^		
**Satisfaction with departmental communication climate**				34.555**	<0.001
Very satisfied	30	9.0	52.53 ± 12.58^a^		
Satisfied	164	49.4	60.80 ± 18.52^b^		
Dissatisfied	125	37.7	78.57 ± 21.28^c^		
Very dissatisfied	13	3.9	81.00 ± 13.58^c^		

Note: *t value; **F value. Values within the same row with different lowercase letters indicate statistically significant differences between groups (*p* < 0.05); values with the same lowercase letter indicate no statistically significant difference (*p* > 0.05)

### Overall descriptive statistics

Normality was assessed using Q-Q plots, and the data were found to be approximately normally distributed. The mean total scores were 67.54 ± 21.33 for missed nursing care, 63.80 ± 14.57 for perceived causes, 40.02 ± 6.54 for moral sensitivity, and 52.62 ± 11.19 for perceived organizational support. Cronbach’s α coefficients ranged from 0.840 to 0.968. The first factor in Harman’s single-factor test explained 22.94% of the variance, which is below the 40% threshold. However, as this test alone is insufficient to fully exclude common method bias, common method bias may still exist due to the self-report nature of the data.

The most frequently missed items were concentrated in psychosocial assessment, early rehabilitation guidance, and muscle strength assessment. High nurse workload was the most commonly reported cause of missed nursing care. The full lists of top five items and reasons are presented in Supplementary Tables 1 and 2.

### Overall correlation analysis

Pearson correlation analysis showed that the total score of missed nursing care among ICU nurses was significantly negatively correlated with the total score of moral sensitivity (*r* = -0.470, *P* < 0.01) and the total score of perceived organizational support (*r* = -0.296, *P* < 0.01). The two dimensions of moral sensitivity, including moral responsibility and strength as well as moral burden, were significantly negatively correlated with the total missed nursing care score and its three content domains (*r* = -0.435 to -0.320, *P* < 0.01). Details are shown in Table 2.

**Table 2:** Correlations between missed nursing care, moral sensitivity, and perceived organizational support among ICU nurses (r value, *N* = 332)

Variables	Missed Nursing Care	Disease Monitoring and Evaluation	Basic Nursing	Specialized Nursing
Moral sensitivity	-0.470**	-0.456**	-0.446**	-0.413**
Moral responsibility and strength	-0.435**	-0.414**	-0.413**	-0.402**
Moral burden	-0.387**	-0.384**	-0.368**	-0.320**
Perceived organizational support	-0.296**	-0.270*	-0.298**	-0.273**

Note: **P<0.01, *P<0.05

### Analysis of influencing factors of missed nursing care

Univariate analysis showed that age, professional title, ICU specialist nurse qualification, ICU type, average number of patients per shift, average monthly overtime hours, satisfaction with human resources, satisfaction with material resources, and satisfaction with communication climate were all associated with missed nursing care scores (*P* < 0.05). Details are shown in Table 1.

Hierarchical multiple linear regression using dummy-coded categorical predictors showed that all three models were statistically significant. Model 1, which included demographic and general work-related characteristics, explained 14.3% of the variance in missed nursing care (R² = 0.143, adjusted R² = 0.111, *P* < 0.001). The addition of job demands in Model 2 significantly increased the explained variance by 12.2% (ΔR² = 0.122, *P* < 0.001), resulting in an R² of 0.266. The addition of job resources in Model 3 further increased the explained variance by 28.2% (ΔR² = 0.282, *P* < 0.001). The final model was statistically significant (F[25,306] = 14.821, *P* < 0.001), explaining 54.8% of the variance in missed nursing care (R² = 0.548; adjusted R² = 0.511).

In the final model, nurses aged < 36 years, female nurses, senior nurses, nurses with ≥ 10 years of ICU experience, and ICU specialist nurses had lower missed nursing care scores relative to their respective reference groups. Monthly overtime of 6–10 and 11–15 h and dissatisfaction with human resources and communication climate were associated with higher missed nursing care scores. Moral responsibility and strength, moral burden, and perceived organizational support were negatively associated with missed nursing care. Other categorical contrasts were not statistically significant. No substantial multicollinearity was detected (VIF range: 1.147–4.652), and the Durbin-Watson statistic was 1.771. Detailed results are presented in Table 3.

**Table 3:** Hierarchical multiple linear regression analysis of factors associated with missed nursing care among ICU nurses (*N* = 332)

Variables	Model 1			Model 2			Model 3		
	B (SE)	β	*P*	B (SE)	β	*P*	B (SE)	β	*P*
**Demographic variables**									
Gender (ref: Male)									
Female	−1.440 (2.884)	−0.027	0.618	−2.527 (2.716)	−0.048	0.353	−4.322 (2.173)	−0.082	0.048
Age (ref: ≥36 years)									
≤ 25 years	−16.256 (6.482)	−0.273	0.013	−15.910 (6.134)	−0.267	0.010	−11.811 (4.896)	−0.198	0.016
26–30 years	−21.205 (4.993)	−0.458	< 0.001	−22.549 (4.742)	−0.487	< 0.001	−13.608 (3.840)	−0.294	< 0.001
31–35 years	−15.824 (3.506)	−0.358	< 0.001	−16.094 (3.328)	−0.364	< 0.001	−10.356 (2.700)	−0.234	< 0.001
Marital status (ref: Unmarried)									
Married	−1.008 (2.809)	−0.024	0.720	−0.696 (2.700)	−0.016	0.797	1.897 (2.189)	0.044	0.387
Education level (ref: Bachelor’s degree or above)									
Junior college or below	−2.078 (2.921)	−0.040	0.477	−2.430 (2.752)	−0.046	0.378	−1.744 (2.218)	−0.033	0.432
Professional title (ref: Nurse-in-charge or above)									
Nurse	−0.161 (5.622)	−0.003	0.977	3.285 (5.297)	0.054	0.536	−8.112 (4.359)	−0.134	0.064
Senior nurse	−4.187 (3.128)	−0.098	0.182	−4.425 (2.939)	−0.104	0.133	−7.072 (2.359)	−0.166	0.003
Type of ICU (ref: General/Emergency ICU)									
Medical ICU	−6.042 (3.058)	−0.111	0.049	−3.561 (3.096)	−0.065	0.251	−1.829 (2.476)	−0.033	0.461
Surgical ICU	4.921 (2.752)	0.101	0.075	6.041 (2.646)	0.124	0.023	3.175 (2.120)	0.065	0.135
Years of ICU experience (ref: 1–4 years)									
5–9 years	2.955 (3.436)	0.059	0.390	1.371 (3.272)	0.028	0.675	−1.695 (2.620)	−0.034	0.518
≥ 10 years	−7.430 (3.877)	−0.159	0.056	−7.972 (3.674)	−0.170	0.031	−6.430 (2.939)	−0.137	0.029
**Job demands**									
Average patient load per shift (ref: 1–2 patients)									
3–4 patients	—	—	—	4.214 (2.708)	0.094	0.121	0.715 (2.197)	0.016	0.745
≥ 5 patients	—	—	—	4.849 (4.547)	0.065	0.287	2.592 (3.642)	0.035	0.477
Average monthly overtime hours (ref: No overtime)									
1–5 h	—	—	—	4.004 (3.222)	0.094	0.215	−0.037 (2.606)	−0.001	0.989
6–10 h	—	—	—	16.271 (3.879)	0.290	< 0.001	9.266 (3.150)	0.165	0.004
11–15 h	—	—	—	20.244 (4.826)	0.272	< 0.001	14.147 (3.886)	0.190	< 0.001
> 15 h	—	—	—	16.214 (4.514)	0.225	< 0.001	3.921 (3.794)	0.054	0.302
**Job resources**									
ICU specialist nurse (ref: No)									
Yes	—	—	—	—	—	—	−11.242 (1.839)	−0.262	< 0.001
Satisfaction with human resource allocation (ref: Satisfied)									
Dissatisfied	—	—	—	—	—	—	6.046 (1.826)	0.138	0.001
Satisfaction with material resource allocation (ref: Satisfied)									
Dissatisfied	—	—	—	—	—	—	3.076 (1.869)	0.070	0.101
Satisfaction with communication climate (ref: Satisfied)									
Dissatisfied	—	—	—	—	—	—	7.182 (1.953)	0.166	< 0.001
Moral responsibility and strength	—	—	—	—	—	—	−0.795 (0.273)	−0.144	0.004
Moral burden	—	—	—	—	—	—	−0.705 (0.286)	−0.119	0.014
Perceived organizational support	—	—	—	—	—	—	−0.193 (0.083)	−0.102	0.021
R²	0.143			0.266			0.548		
Adjusted R²	0.111			0.223			0.511		
ΔR²	0.143			0.122			0.282		
F change	4.448			8.693			27.251		
P for F change	< 0.001			< 0.001			< 0.001		
Overall F	4.448			6.292			14.821		
Overall model P	< 0.001			< 0.001			< 0.001		

Note: B = unstandardized regression coefficient; SE = standard error; β = standardized regression coefficient; Ref = reference category. Model 1 included demographic and general work-related characteristics; Model 2 additionally included job demands; Model 3 additionally included job resources. Categorical predictors were dummy-coded

### Results of latent profile analysis of missed nursing care among ICU nurses

For the LPA, the mean scores of the three content domains, rather than individual items, were used as observed indicators. First, the original scale developers noted that Part A of the scale consists of relatively independent nursing activities without predefined subdimensions suitable for item-level factor analysis, but it is divided into three content domains. Second, using domain mean scores reduces model complexity and improves parameter estimate stability, as LPA with a large number of indicators can lead to convergence difficulties and unstable solutions. The three content domains also demonstrated satisfactory internal consistency in this study (Cronbach’s α ranging from 0.894 to 0.931). Accordingly, mean scores of each content domain were calculated and used as input variables for LPA, as shown in Table 4.

**Table 4:** Number of items and reliability of each content domain

Content domain	Number of items	Content domain score (x̅±s)	Cronbach’s Alpha
Disease Monitoring and Evaluation	18	33.88 ± 10.22	0.931
Basic Nursing	13	22.36 ± 7.68	0.928
Specialized Nursing	6	11.30 ± 4.69	0.894

Using the mean scores of the three content domains of Part A of the ICU Missed Nursing Care Scale (disease monitoring and evaluation, basic nursing care, and specialized nursing care) as observed indicators, models with 1 to 5 latent profiles were fitted sequentially (Table 5). Considering statistical fit indices, class size, model parsimony, and substantive interpretability, the 3-profile model was selected as the most appropriate solution. Although the AIC, BIC, and aBIC values continued to decrease from the three-profile to the five-profile solutions, the magnitude of these decreases diminished substantially after the three-profile model, suggesting diminishing returns in model fit improvement. More importantly, the four-profile solution contained an extremely small latent class (0.9%, *n* = 3), and the five-profile solution also contained a negligible class (0.9%, *n* = 3). Such small classes may provide insufficiently stable and representative estimates and may lead to unstable parameter estimates [22]. In addition, the LMR test was not significant for the four-profile (*p* = 0.0537) or five-profile (*p* = 0.2596) solutions, indicating that adding a fourth or fifth profile did not significantly improve model fit. In contrast, the three-profile model demonstrated adequate classification accuracy (Entropy = 0.925), significant LMR (*P* = 0.0275) and BLRT (*P* < 0.001), without extremely small latent classes (smallest class: 15.06%, *n* = 50), and clear substantive interpretability and theoretical coherence, with the three profiles representing low, moderate, and high levels of missed nursing care. The posterior probabilities ranged from 94.5% to 98.0% (Table 6).

**Table 5:** Indices of latent profile analysis of missed nursing care among ICU nurses

Model	k	log(L)	AIC	BIC	aBIC	Entropy	LMR	BLRT	Class probability
1	6	-967.464	1946.928	1969.759	1950.726				100%
2	10	-648.735	1317.471	1355.522	1323.802	0.920	0.0016	0.0000	68.98%/31.02%
3*	14	-524.122	1076.244	1129.516	1085.107	0.925	0.0275	0.0000	56.63%/15.06%/28.31%
4	18	-472.182	980.363	1048.856	991.759	0.943	0.0537	0.0000	28.31%/14.76%/56.03%/0.9%
5	22	-439.196	922.391	1006.104	936.319	0.910	0.2596	0.0000	47.89%/12.95%/19.28%/18.98%/0.9%

Note: LL = log likelihood; AIC = Akaike Information Criterion; BIC = Bayesian Information Criterion; aBIC = adjusted Bayesian Information Criterion; LMR = Lo-Mendell-Rubin likelihood ratio test (K − 1 vs. K classes); BLRT = bootstrap likelihood ratio test (K − 1 vs. K classes); * = best-fitting model

**Table 6:** Average posterior probabilities of each latent profile

Latent Profile	1	2	3
1	0.980	0.000	0.020
2	0.000	0.958	0.042
3	0.046	0.009	0.945

**Fig. 1:**
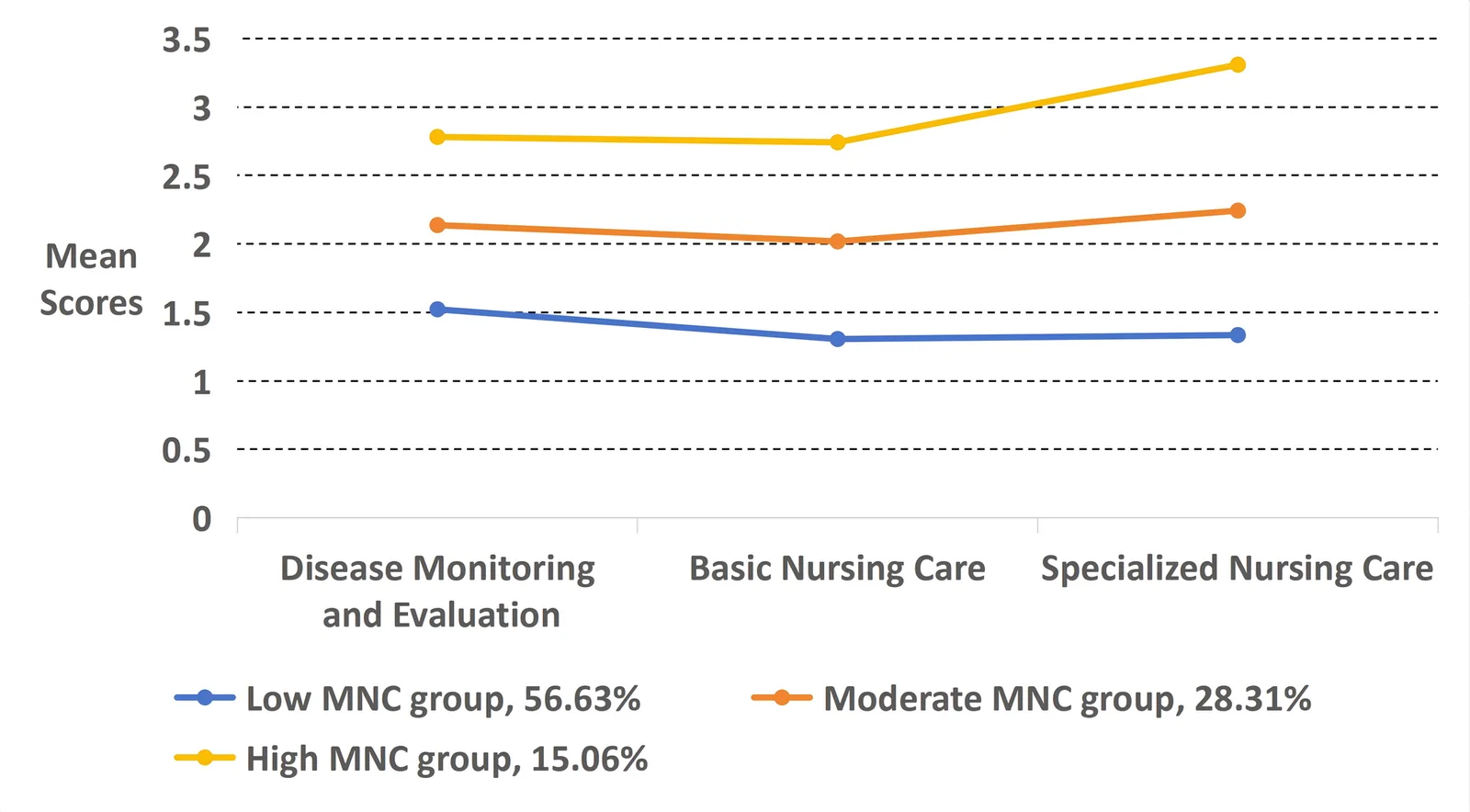
Latent profile analysis of missed nursing care among ICU nurses

The three profiles were labeled as follows: low missed nursing care group (188 cases, 56.63%), moderate missed nursing care group (94 cases, 28.31%), and high missed nursing care group (50 cases, 15.06%). Differences in scores among the three profiles across all three dimensions were statistically significant (*P* < 0.001), with scores ranked in descending order as high missed nursing care group > moderate missed nursing care group > low missed nursing care group (Fig. 1).

### Comparison of characteristics of ICU nurses among different latent profiles

Chi-square tests were used for univariate analysis of demographic characteristics among ICU nurses in different latent profiles. The results showed statistically significant differences among the three profiles in age, professional title, ICU specialist nurse qualification, type of ICU, average number of patients per shift, average monthly overtime hours, satisfaction with departmental human resource allocation, satisfaction with departmental material resource allocation, and satisfaction with departmental communication climate (*P* < 0.05), whereas no significant differences were found in gender, marital status, education level, or working years in the ICU (*P* > 0.05). Details are shown in Table 7.

**Table 7:** Distribution of demographic variables among ICU nurses in different latent profiles (*N* = 332)

Demographic Variable	Low MNC Group (*n* = 188)	Moderate MNC Group (*n* = 94)	High MNC Group (*n* = 50)	χ²	*P*-value
**Gender**				0.266	0.875
Male	40(21.3%)	19(20.2%)	9(18.0%)		
Female	148(78.7%)	75(79.8%)	41(82.0%)		
**Age (years)**				40.708	<0.001
≤ 25	27(14.4%)	20(21.3%)	3(6.0%)		
26–30	71(37.8%)	22(23.4%)	8(16.0%)		
31–35	70(37.2%)	34(36.2%)	17(34.0%)		
36–40	18(9.6%)	15(16.0%)	21(42.0%)		
≥ 41	2(1.1%)	3(3.2%)	1(2.0%)		
**Marital status**				5.744	0.057
Married	93(49.5%)	47(50.0%)	34(68.0%)		
Unmarried	95(50.5%)	47(50.0%)	16(32.0%)		
**Education level**				8.477	0.205
Technical secondary school	3(1.6%)	1(1.1%)	1(2.0%)		
Junior college	40(21.3%)	21(22.3%)	3(6.0%)		
Bachelor’s degree	143(76.1%)	69(73.4%)	45(90.0%)		
Master’s degree	2(1.1%)	3(3.2%)	1(2.0%)		
**Professional title**				28.286	<0.001
Nurse	27(14.4%)	20(21.3%)	1(2.0%)		
Senior nurse	108(57.4%)	45(47.9%)	19(38.0%)		
Nurse-in-charge	51(27.1%)	29(30.9%)	30(60.0%)		
Vice supervisor nurse	2(1.1%)	0(0.0%)	0(0.0%)		
**ICU specialist nurse qualification**				50.688	<0.001
Yes	115(61.2%)	23(24.5%)	9(18.0%)		
No	73(38.8%)	71(75.5%)	41(82.0%)		
**Type of ICU**				20.692	0.002
Medical ICU	44(23.4%)	11(11.7%)	7(14.0%)		
Surgical ICU	42(22.3%)	23(24.5%)	20(40.0%)		
General ICU	99(52.7%)	51(54.3%)	21(42.0%)		
Emergency ICU	3(1.6%)	9(9.6%)	2(4.0%)		
**Working years in ICU (years)**				5.248	0.263
1–4	94(50.0%)	45(47.9%)	16(32.0%)		
5–9	43(22.9%)	22(23.4%)	15(30.0%)		
≥ 10	51(27.1%)	27(28.7%)	19(38.0%)		
**Average number of patients per shift**				13.103	0.011
1–2	59(31.4%)	19(20.2%)	4(8.0%)		
3–4	114(60.6%)	66(70.2%)	40(80.0%)		
≥ 5	15(8.0%)	9(9.6%)	6(12.0%)		
**Average monthly overtime hours (hours)**				36.140	<0.001
No overtime	41(21.8%)	11(11.7%)	2(4.0%)		
1–5	100(53.2%)	41(43.6%)	17(34.0%)		
6–10	23(12.2%)	22(23.4%)	13(26.0%)		
11–15	14(7.4%)	6(6.4%)	10(20.0%)		
>15	10(5.3%)	14(14.9%)	8(16.0%)		
**Satisfaction with departmental human resource allocation**				76.299	<0.001
Very satisfied	10(5.3%)	0(0.0%)	0(0.0%)		
Satisfied	96(51.1%)	13(13.8%)	10(20.0%)		
Dissatisfied	79(42.0%)	70(74.5%)	28(56.0%)		
Very dissatisfied	3(1.6%)	11(11.7%)	12(24.0%)		
**Satisfaction with departmental material resource allocation**				54.597	<0.001
Very satisfied	26(13.8%)	0(0.0%)	0(0.0%)		
Satisfied	75(39.9%)	13(13.8%)	14(28.0%)		
Dissatisfied	82(43.6%)	70(74.5%)	31(62.0%)		
Very dissatisfied	5(2.7%)	11(11.7%)	5(10.0%)		
**Satisfaction with departmental communication climate**				114.656	<0.001
Very satisfied	26(13.8%)	4(4.3%)	0(0.0%)		
Satisfied	130(69.1%)	16(17.0%)	18(36.0%)		
Dissatisfied	31(16.5%)	65(69.1%)	29(58.0%)		
Very dissatisfied	1(0.5%)	9(9.6%)	3(6.0%)		

Statistically significant differences were found across the three profiles in all scale scores except the absolute nursing shortage subscale (*p* = 0.052) (Table 8). Post hoc comparisons (LSD-t test) revealed profile-specific pairwise differences across the measured variables, although not all pairwise comparisons were statistically significant. Specifically, the low group was characterized by the lowest missed nursing care scores, the highest moral sensitivity, and the highest perceived organizational support, along with the highest proportion of ICU specialist nurses; the moderate group exhibited the lowest moral sensitivity; and the high group had the highest missed nursing care scores and the lowest perceived organizational support, with the highest rates of surgical ICU nurses and overtime hours. Details are shown in Table 8.

**Table 8:** Descriptive analysis of missed nursing care items, perceived causes of missed nursing care, moral sensitivity, and perceived organizational support among ICU nurses across different latent profiles (*N* = 332, mean ± SD)

Profile	Low MNC Group (*n* = 188)	Moderate MNC Group (*n* = 94)	High MNC Group (*n* = 50)	F	*P*-value
Missed nursing care	52.28 ± 7.75^a^	77.98 ± 8.02^b^	105.28 ± 12.92^c^	811.222	< 0.001
Disease monitoring and evaluation	27.35 ± 5.80^a^	38.41 ± 5.56^b^	49.9 ± 7.34^c^	317.282	< 0.001
Basic nursing care	16.96 ± 2.83^a^	26.13 ± 3.50^b^	35.56 ± 5.23^c^	470.991	< 0.001
Specialized nursing care	7.96 ± 1.63^a^	13.44 ± 2.10^b^	19.82 ± 2.15^c^	781.455	< 0.001
Causes of missed nursing care	63.67 ± 15.32^b^	60.19 ± 12.66^b^	71.10 ± 12.43^a^	9.645	<0.001
Human resource factors	19.56 ± 4.12^b^	19.01 ± 4.16^b^	21.36 ± 3.71^a^	5.607	0.004
Absolute shortage of nursing staff	9.62 ± 2.01^a^	9.59 ± 2.13^a^	10.36 ± 1.75^a^	2.983	0.052
Relative shortage of nursing staff	9.94 ± 2.99^ab^	9.43 ± 3.08^b^	11.00 ± 2.70^a^	4.583	0.011
Material resource factors	10.41 ± 3.25^ab^	9.66 ± 2.73^b^	11.38 ± 2.82^a^	6.338	0.002
Communication factors	14.00 ± 4.30^b^	13.14 ± 3.64^b^	15.80 ± 4.06^a^	6.937	0.001
Organizational management factors	19.70 ± 6.21^b^	18.38 ± 5.11^b^	22.56 ± 5.04^a^	11.066	<0.001
Moral sensitivity	43.21 ± 5.85^a^	34.80 ± 4.23^b^	37.84 ± 5.28^c^	82.227	<0.001
Moral responsibility and strength	25.47 ± 3.38^a^	20.96 ± 3.14^b^	22.34 ± 3.11^c^	64.642	<0.001
Moral burden	17.74 ± 3.34^a^	13.84 ± 2.69^b^	15.50 ± 3.34^c^	49.307	<0.001
Perceived organizational support	55.23 ± 11.37^a^	50.67 ± 11.17^b^	46.48 ± 6.67^c^	24.331	<0.001

Note: Different letters indicate statistically significant differences between groups based on post hoc comparisons (*P* < 0.05)

The top five missed nursing care items differed noticeably across the three profiles: the low missed nursing care group was dominated by basic assessment and psychological care items (1.68–1.90), the moderate missed nursing care group involved condition assessment and care planning (2.44–2.79), and the high missed nursing care group included core clinical nursing items such as sedation and analgesia management (3.30–4.30). High workload of nurses was the most reported primary cause across all three profiles (3.79–3.86). The high missed nursing care group had the highest score for perceived excessive nursing interruptions (3.30). The detailed lists of top five items and reasons are presented in Supplementary Tables 3 and 4.

### Correlation analysis between missed nursing care, moral sensitivity and perceived organizational support among ICU nurses in different profiles

The correlation between moral sensitivity and missed nursing care varied across different latent profiles. In the low missed nursing care group, moral sensitivity and its two dimensions were significantly negatively correlated with the total missed nursing care score (*r* = -0.299 to -0.241, *P* < 0.01), and most correlations with the three content domains were also statistically significant. No significant correlation was found in the moderate missed nursing care group. In the high missed nursing care group, only moral burden was significantly negatively correlated with basic nursing care (*r* = -0.284, *P* < 0.05). The total missed nursing care score and its three content domains showed no significant correlation with perceived organizational support in any profile (*P* > 0.05), as shown in Table 9.

**Table 9:** Correlations of missed nursing care with moral sensitivity and perceived organizational support among ICU nurses across different latent profiles

Profile	Variable	Missed Nursing Care	Disease Monitoring and Evaluation	Basic Nursing Care	Specialized Nursing Care
Low MNC Group (*n* = 188)	Moral sensitivity	-0.299**	-0.248**	-0.191**	-0.210**
	Moral responsibility and strength	-0.241**	-0.184**	-0.154*	-0.224**
	Moral burden	-0.280**	-0.247**	-0.179*	-0.141
	Perceived organizational support	-0.060	-0.050	-0.045	-0.030
Moderate MNC Group (*n* = 94)	Moral sensitivity	-0.089	-0.157	0.006	0.066
	Moral responsibility and strength	-0.112	-0.128	-0.046	-0.013
	Moral burden	-0.009	-0.097	0.063	0.119
	Perceived organizational support	-0.073	-0.021	-0.135	0.001
High MNC Group (*n* = 50)	Moral sensitivity	-0.142	-0.150	-0.215	0.184
	Moral responsibility and strength	-0.031	-0.077	-0.060	0.223
	Moral burden	-0.195	-0.166	-0.284*	0.084
	Perceived organizational support	-0.200	-0.222	-0.182	0.002

Note: **P<0.01, *P<0.05

### Multinomial logistic regression analysis of factors associated with latent profile membership

Multinomial logistic regression was used to examine factors associated with latent profile membership. The moderate MNC group was selected as the reference category to facilitate direct comparisons with both the low- and high-MNC profiles. This choice was made for analytical interpretability and does not imply that the moderate profile represents a temporal transitional stage. Demographic and work-related variables showing statistically significant differences across profiles in the univariate comparisons (Table 7) were entered into the model, together with moral responsibility and strength, moral burden, and perceived organizational support. Categorical predictors were coded using the categories and reference groups specified in the Statistical Analysis section, and the same dichotomous coding of the three satisfaction variables as that used in the hierarchical regression was applied.

**Table 10:** Multinomial logistic regression analysis of factors associated with latent profile membership among ICU nurses

Item		B	SE		Wald χ²		*P*-value		OR	95%CI	
**Low MNC Group vs. Moderate MNC Group**											
Moral responsibility and strength		0.246	0.076		10.523		0.001		1.279	1.102–1.484	
Moral burden		0.281	0.081		12.105		0.001		1.324	1.130–1.551	
Perceived organizational support		-0.006	0.024		0.052		0.820		0.995	0.948–1.043	
Age (ref: ≥36 years)											
≤ 25 years		1.518	1.251		1.472		0.225		4.562	0.393–52.946	
26–30 years		1.839	0.903		4.147		0.042		6.292	1.072–36.941	
31–35 years		1.026	0.743		1.907		0.167		2.790	0.650-11.967	
Professional title (ref: nurse-in-charge or above)											
Nurse		0.437	1.242		0.124		0.725		1.548	0.136–17.648	
Senior nurse		0.498	0.664		0.564		0.453		1.646	0.448–6.044	
ICU specialist nurse (ref: No)											
Yes		2.044	0.545		14.063		< 0.001		7.718	2.652–22.459	
Type of ICU (ref: General/emergency ICU)											
Medical ICU		1.431	0.743		3.705		0.054		4.181	0.974–17.948	
Surgical ICU		0.504	0.579		0.759		0.384		1.656	0.532–5.150	
Average patient load per shift (ref: 1–2 patients)											
3–4 patients		-0.226	0.617		0.134		0.714		0.798	0.238–2.674	
≥ 5 patients		-1.418	1.035		1.879		0.170		0.242	0.032–1.840	
Average monthly overtime hours (ref: No overtime)											
1–5 h		-0.273	0.812		0.113		0.737		0.761	0.155–3.737	
6–10 h		-1.557	0.951		2.679		0.102		0.211	0.033–1.360	
11–15 h		-1.035	1.171		0.781		0.377		0.355	0.036–3.524	
> 15 h		-0.348	1.115		0.097		0.755		0.706	0.079–6.288	
Satisfaction (ref: Satisfied)											
Human resource (Dissatisfied)		-2.211	0.536		16.986		< 0.001		0.110	0.038–0.314	
Material resource (Dissatisfied)		-2.316	0.551		17.643		< 0.001		0.099	0.033–0.291	
Communication climate (Dissatisfied)		-2.637	0.510		26.787		< 0.001		0.072	0.026–0.194	
**High MNC Group vs. Moderate MNC Group**											
Moral responsibility and strength		0.078	0.072		1.152		0.283		1.081	0.938–1.246	
Moral burden		0.235	0.080		8.516		0.004		1.265	1.080–1.481	
Perceived organizational support		-0.070	0.023		8.967		0.003		0.932	0.890–0.976	
Age (ref: ≥36 years)											
≤ 25 years		-1.328	1.211		1.203		0.273		0.265	0.025–2.844	
26–30 years		-1.249	0.797		2.458		0.117		0.287	0.060–1.367	
31–35 years		-1.291	0.550		5.519		0.019		0.275	0.094–0.807	
Professional title (ref: nurse-in-charge or above)											
Nurse		-2.436	1.582		2.370		0.124		0.088	0.004–1.945	
Senior nurse		-0.731	0.556		1.728		0.189		0.481	0.162–1.432	
ICU specialist nurse (ref: No)											
Yes		-1.052	0.551		3.646		0.056		0.349	0.119–1.028	
Type of ICU (ref: General/emergency ICU)											
Medical ICU		1.415	0.790		3.210		0.073		4.118	0.875–19.374	
Surgical ICU		0.953	0.529		3.250		0.071		2.593	0.920–7.307	
Average patient load per shift (ref: 1–2 patients)											
3–4 patients		-0.476	0.778		0.374		0.541		0.622	0.135–2.855	
≥ 5 patients		-1.106	1.091		1.028		0.311		0.331	0.039–2.808	
Average monthly overtime hours (ref: No overtime)											
1–5 h		0.481	1.056		0.207		0.649		1.617	0.204–12.812	
6–10 h		1.089	1.103		0.974		0.324		2.974	0.342–25.839	
11–15 h		2.883	1.288		5.011		0.025		17.868	1.431-223.051	
> 15 h		1.555	1.180		1.738		0.187		4.737	0.469–47.853	
Satisfaction (ref: Satisfied)											
Human resource (Dissatisfied)		-0.504	0.586		0.739		0.390		0.604	0.192–1.906	
Material resource (Dissatisfied)		-1.062	0.558		3.616		0.057		0.346	0.116–1.033	
Communication climate (Dissatisfied)		-0.980	0.505		3.767		0.052		0.375	0.139–1.010	

Note: B = unstandardized multinomial logistic regression coefficient; SE = standard error; OR = odds ratio; CI = confidence interval. The moderate MNC group was the reference outcome category. Reference categories for categorical predictors are indicated in the table

The multinomial logistic regression model was statistically significant (χ² = 360.497, *P* < 0.001), with Cox-Snell, Nagelkerke, and McFadden pseudo-R² values of 0.662, 0.775, and 0.563, respectively. Compared with the moderate MNC group, higher moral responsibility and strength, higher moral burden, age 26–30 years, and ICU specialist nurse qualification were positively associated with membership in the low MNC group, whereas dissatisfaction with human resources, material resources, and communication climate was negatively associated with membership in the low MNC group. Compared with the moderate MNC group, higher moral burden, lower perceived organizational support, age 31–35 years, and 11–15 h of monthly overtime were associated with membership in the high MNC group. Professional title and average patient load per shift were not significantly associated with profile membership after adjustment. Detailed results are presented in Table 10.

## Discussion

A total of 332 ICU nurses were included in this study. The results showed that the total score of missed nursing care was 67.54 ± 21.33, which was slightly higher than the survey results by Zhou et al. [8] in China. Given differences in measurement tools used in different studies, direct comparison of absolute scores is of limited significance. However, consistent with the overall trend, missed nursing care in ICUs is a prevalent global problem. A systematic review and Meta-analysis covering 14 countries and more than 20,000 nurses showed that the global reported rate of missed nursing care ranges from 55% to 98% [23]. Another study on ICU nurses found that the score of missed nursing care was 38.47 ± 13.82 (using the MISSCARE scale), further confirming that missed nursing care is widespread in different countries and medical systems [24].

In terms of specific missed items, the items with the highest incidence of missed nursing care in this study were concentrated in non-technical and supportive nursing activities such as psychosocial assessment (2.39 points), early rehabilitation guidance (2.38 points), and psychological care (2.17 points). This is consistent with a systematic review by Imam et al. [25], which focused on acute care institutions in low- and middle-income countries. It pointed out that the most frequently missed nursing activities by nurses are non-clinical tasks, such as comfort care and communication care. When faced with time and resource constraints, nurses typically prioritize life-saving technical procedures. They tend to delay non-urgent, non-technical nursing tasks. This forms a nursing priority pattern that is prevalent across different cultures [26]. These findings suggest that non-technical and humanistic nursing contents are high-incidence areas of missed nursing care in ICUs. Therefore, we should promote the balanced development of ICU nursing practice toward both technology and humanism. We should integrate supportive care needs into routine nursing processes and improve nurses’ humanistic care ability and soft skills to enhance their core competence from a flexible and humanistic perspective.

In terms of influencing factors, the hierarchical regression analysis showed that monthly overtime of 6–10 and 11–15 h and dissatisfaction with human resource allocation and communication climate were associated with higher missed nursing care, whereas ICU specialist nurse qualification, moral responsibility and strength, moral burden, and perceived organizational support were associated with lower missed nursing care. Several demographic and professional characteristics, including age, sex, professional title, and ICU experience, were also associated with missed nursing care. These findings are broadly consistent with the factors discussed by Siela [27]. The overall correlation analysis also showed a negative association between moral sensitivity and missed nursing care among Chinese ICU nurses, and the relationship between moral sensitivity and missed nursing care has also been examined in previous research [28]. In summary, reducing missed nursing care in ICUs may require attention to both organizational conditions and individual resources. On the one hand, nursing scheduling and human resource allocation should be optimized to reduce workload. On the other hand, moral sensitivity training should be included in ICU nurses’ professional development. Efforts should also be made to strengthen perceived organizational support and establish a supportive practice environment. These strategies may help address missed nursing care and could contribute to patient safety and nursing quality, although their effectiveness requires further evaluation.

Based on the JD-R theoretical framework, this study used LPA to identify the group heterogeneity of missed nursing care among ICU nurses. The analysis identified three distinct profiles characterized by progressively higher levels of missed nursing care. The low group accounted for the highest proportion (56.63%), suggesting that more than half of the nurses maintained relatively complete and standardized nursing practice. The moderate group accounted for 28.31%, while the high group (15.06%) showed the most severe care deficiencies, potentially indicating greater concerns regarding patient safety. The three subgroups also showed characteristic differences in the structure of missed items. The low group mainly omitted non-technical and supportive nursing items, while the moderate group extended deficiencies to condition assessment and care planning, and the high group showed deficits in core clinical nursing items directly related to patient safety. The three profiles also differed in the distribution of missed-care activities. The low profile was characterized mainly by missed non-technical and supportive nursing activities, whereas the moderate and high profiles showed broader involvement of assessment, care planning, and core clinical nursing activities. These cross-sectional differences indicate distinct patterns across profiles but should not be interpreted as evidence of temporal progression from one profile to another. A Meta-synthesis by Fan et al. [29] also showed that ICU missed nursing care is predictable and non-random. The risk of missed nursing care varies among different subgroups, making it difficult to achieve effective control through a one-size-fits-all strategy. Therefore, clinical managers may consider implementing stratified and targeted interventions based on latent profile characteristics, although the effectiveness of such approaches requires further evaluation in future studies. For the low missed nursing care group, focus could be placed on maintaining resource supply, optimizing work processes, and preventing burnout. For the moderate missed nursing care group, one may consider strengthening ethical education, improving moral sensitivity, and enhancing organizational support to address factors associated with missed nursing care. For the high missed nursing care group, the intervention focus could be on prioritizing reducing workload and standardizing staffing to ensure delivery of core nursing interventions, followed by competency and behavioral improvement strategies.

In this study, nurses in the low missed nursing care group were less likely to report dissatisfaction with departmental human resource allocation, material resource allocation, and communication climate compared with the moderate group (Table 10). The descriptive data (Table 8) also showed that the low group had the highest perceived organizational support score among the three groups (55.23 ± 11.37). This pattern may be partly related to the fact that the low group was predominantly composed of ICU specialist nurses (61.2%). These nurses possess stronger clinical judgment, process coordination, and risk response capabilities [30]. In the high-workload and resource-constrained ICU environment, higher professional competence helps offset the adverse effects of insufficient resources and enables nurses to maintain relatively complete nursing practice [31]. At the same time, ICU specialist nurses may hold higher self-requirements for nursing quality and stricter evaluation standards for the work environment. The tension between their high professional standards and perceived system insufficiency is likely to affect satisfaction evaluation through an expectation-reality gap mechanism [32, 33]. Thus, professional competence may also play an important role in maintaining nursing quality alongside subjective satisfaction. When evaluating nursing quality, nursing managers should not rely solely on satisfaction as a judgment criterion, but rather conduct a comprehensive assessment combining professional competence and nursing behavior performance.

The results of this study showed that ICU specialist nurse qualification was significantly associated with membership in the low missed nursing care subgroup (OR = 7.718, *P* < 0.001). The proportion of ICU specialist nurses was as high as 61.2% in the low missed nursing care group, but only 18.0% in the high missed nursing care group, suggesting that specialized competence may be associated with more complete nursing care. From the perspective of the JD-R model, specialist nurse qualification belongs to a high-level individual resource. Compared with non-specialist nurses, ICU specialist nurses have received systematic training in critical care pathophysiology, assessment techniques, emergency treatment, and humanistic care. They may be better equipped to plan work, manage key nursing nodes, and reduce omissions in an environment with high workload, tight manpower, and frequent emergencies [34], potentially helping them cope with high job demands. However, the potential role of specialist nurse qualification may also depend on individual ability, organizational resources, and work environment [35, 36]. In future nursing management, training and certification of ICU specialist nurses may serve as a key strategy for addressing missed nursing care, though this requires confirmation in future intervention studies. ICUs should encourage and support nurses to obtain specialist certification, thereby improving professional competence and strengthening resilience in coping with work pressure. In clinical practice, the potential benefits of specialist nurses in high‑pressure settings could be further supported through clear role definition and institutional support, though causality cannot be inferred due to the cross-sectional design.

Different patterns of association between moral sensitivity and missed nursing care were observed across profiles, suggesting profile-specific heterogeneity in these associations. In the low missed nursing care group, moral sensitivity and its subdimensions were significantly negatively correlated with missed nursing care. Nurses in this group had the highest moral sensitivity and were mostly specialist nurses, suggesting that the combination of high ethical awareness and professional competence may contribute to maintaining nursing care under pressure. This may help nurses to prioritize patient care even under pressure, suggesting that moral sensitivity may be a resource associated with lower missed nursing care. In the moderate missed nursing care group, moral sensitivity was the lowest and showed no significant correlation with missed nursing care. Nurses in this group had the lowest moral sensitivity and a lower level of perceived organizational support than the low missed nursing care group, possibly indicating fewer individual and organizational resources. Relevant studies have pointed out that when nurses lack both internal moral motivation and sufficient organizational support, moral sensitivity is difficult to translate into actual nursing behavior, and its behavior regulation role may be significantly weakened or even lost [37, 38]. In the high missed nursing care group, nurses experienced relatively high work demands and frequent overtime, with the most severe levels of missed nursing care. Under these high job demands, only moral burden showed a significant negative correlation with basic nursing care, while no other variables were significantly correlated. This may indicate that the association between moral sensitivity and missed nursing care was less evident under higher job demands. Previous studies have also reported associations between high workload and moral sensitivity, suggesting that the role of ethical resources may vary under demanding clinical conditions [39, 40]. Therefore, the role of ethical resources should be considered together with workload and other contextual factors.

The distinction between the two dimensions of moral sensitivity helps explain the heterogeneous associations of moral burden with missed nursing care across subgroups. Moral responsibility and strength reflect nurses’ active ethical engagement, including the willingness to recognize moral obligations and take responsibility for patient care. In contrast, moral burden reflects the psychological cost of ethical awareness, referring to the distress nurses experience when they perceive patients’ suffering or feel unable to fulfill their ethical duties due to contextual constraints [19]. Thus, higher scores on moral responsibility and strength indicate greater ethical capacity, whereas higher scores on moral burden indicate greater emotional reactivity to ethical challenges [20]. This conceptual distinction clarifies the differential associations of moral burden across the three profiles. In the low missed nursing care group, predominantly composed of specialist nurses with high professional competence, higher moral burden may reflect heightened ethical sensitivity and awareness of patient needs, which may be associated with greater attention to patient care. In the moderate missed nursing care group, no significant correlation was found, possibly reflecting ethical numbing or disengagement under moderate workload with limited organizational support. In the high missed nursing care group, moral burden remained significantly associated with basic nursing care, while other associations with moral sensitivity were less evident under high job demands; only the distress component of moral sensitivity remained detectable, while the positive motivational component may have been less apparent under high job demands. This pattern is consistent with the conceptualization of moral burden as a distress-based rather than a motivation-based construct. It may serve as a catalyst for proactive care only when nurses possess sufficient professional competence and organizational resources to act on their ethical awareness; otherwise, it remains a passive emotional response to unaddressed ethical challenges.

The above findings suggest profile-specific patterns in the associations between moral sensitivity and missed nursing care. These cross-sectional differences should not be interpreted as evidence of a nonlinear effect because nonlinearity or statistical interactions were not formally tested. Rather, the association between moral sensitivity and missed nursing care may vary according to contextual factors such as workload, organizational support, and professional competence. Under conditions of high job demands and limited organizational resources, the association between moral sensitivity and lower missed nursing care may become less apparent. Therefore, efforts to strengthen moral sensitivity alone may be insufficient unless accompanied by improvements in workload management and organizational support [41]. These findings highlight the importance of considering both individual ethical resources and the broader work environment when addressing missed nursing care.

In terms of perceived organizational support, correlation analysis of the total sample in this study showed that perceived organizational support was significantly negatively correlated with missed nursing care (*r* = -0.296, *P* < 0.01). This suggests that higher levels of perceived organizational support are associated with lower levels of missed nursing care, which is consistent with previous research findings [42]. However, in subgroup correlation analysis based on LPA, the association between perceived organizational support and missed nursing care was not statistically significant. This indicates that within homogeneous subgroups, the association between perceived organizational support and missed nursing care may be weakened or overshadowed by other factors. Further multinomial logistic regression revealed that perceived organizational support was significantly associated with membership in the high missed nursing care group relative to the moderate group. Specifically, lower perceived organizational support was associated with a higher likelihood of being classified into the high missed nursing care group. This suggests that the association between perceived organizational support and missed nursing care may not be consistent across all groups, but instead shows subgroup specificity and context dependence. Compared with the low and moderate missed nursing care groups, the high missed nursing care group faced a heavier workload, longer overtime hours. In such settings with high job demands and scarce external resources, greater organizational support may be particularly relevant to the high MNC profile of missed nursing care. Kang et al. [43] also noted that in understaffed clinical settings, organizational support serves as a core buffering variable against missed nursing care resulting from role overload. The non‑significant association between perceived organizational support and missed nursing care in the low and moderate subgroups may be explained as follows. In the low missed nursing care group, individual resources such as moral sensitivity and professional competence may be particularly relevant, which may partly explain the non-significant association between perceived organizational support and missed nursing care. A study by Tsegaye et al. [44] on missed nursing care among Ethiopian obstetric nurses also identified working experience as a factor associated with missed nursing care, suggesting that individual professional characteristics may be relevant when considering missed nursing care. On the other hand, the impact of perceived organizational support on missed nursing care may mainly take an indirect path [45], such as through teamwork, professional resilience, and moral sensitivity, rather than a simple direct association. In summary, the association between perceived organizational support and missed nursing care may vary according to work demands, individual characteristics, and professional resources. The strength of this association may show context dependence and may be more pronounced in high-workload and high-risk clinical settings. This finding reminds nursing managers that intervention focuses and strategies regarding perceived organizational support should differ for nurses with distinct characteristics. Strengthening organizational support and resource provision for high‑workload and high‑risk groups may help address missed nursing care in this population, though this requires testing in future intervention studies.

Based on the above results, this study proposes targeted intervention strategies for different profiles. For the low missed nursing care group (56.63%), which was characterized by a low level of missed nursing care and a relatively high proportion of ICU specialist nurses, the intervention focus could be on maintaining favorable working conditions and reducing unnecessary workload. Nursing managers may consider optimizing resource allocation and reducing non-nursing tasks to help maintain the relatively low level of missed nursing care and prevent burnout. For the moderate missed nursing care group (28.31%), which was characterized by the lowest moral sensitivity and moderate perceived organizational support, intervention strategies could focus on strengthening moral sensitivity and organizational support. Ethics training, case discussions, reflective practice, and supervision may be considered, together with measures to improve team communication and emotional support, thereby addressing factors associated with higher levels of missed nursing care. For the high missed nursing care group (15.06%), which had the highest level of missed nursing care and relatively high work demands, the intervention focus could prioritize reducing workload, controlling overtime, optimizing staffing and nurse-to-patient ratios, reducing nursing interruptions, streamlining processes, and improving the communication climate. Workload management may provide an important context for moral education and organizational support to play a role.

### Limitations

The results of this study are consistent with existing studies and have further expansions. Multiple studies have pointed out that ICU missed nursing care is a problem that needs attention and solution, and moral sensitivity and perceived organizational support are negatively correlated with missed nursing care, which is consistent with the conclusions of this study. However, this study conducted subgroup analysis on the level of missed nursing care among ICU nurses by using the LPA method, which provides certain reference value for clinical management. This study strived for scientific rigor in method design and data analysis, but still had the following limitations: First, a cross-sectional design was adopted, which precludes causal relationships. Second, convenience sampling was used, and the sample was only from 5 tertiary Grade A hospitals in Sichuan Province, which may have selection bias and limits the generalizability of the results. Additionally, participants were recruited from five tertiary hospitals, and nurses within the same hospital may share organizational characteristics that could violate the independence assumption. We did not adjust for hospital-level clustering due to the limited number of hospitals (*n* = 5). Future studies with a larger number of hospitals should consider multilevel modeling to address this clustering effect. Third, self-reported data were used, which may be subject to recall bias and social desirability bias. Although the first factor in Harman’s single-factor test explained only 22.94% of the variance, suggesting that common method bias was unlikely to be a major concern, this test alone cannot fully exclude its presence. Common method bias may therefore still exist due to the self-report nature of the data. Future studies could incorporate objective measures or multi-source data to further reduce this bias. Fourth, the study lacked objective patient safety or care quality indicators, relying instead on nurses’ self-reported perceptions. Fifth, the sample size of the high missed nursing care group was small (*n* = 50), and subgroup analysis should be interpreted with caution. Future studies could adopt a longitudinal design, multi-center large samples, and integrate self-reported data, objective management indicators, and patient safety outcomes to further verify the findings of this study.

## Conclusion

Using LPA, this study identified three distinct and interpretable profiles of missed nursing care among ICU nurses, characterized by low, moderate, and high levels of missed nursing care. The profiles differed in missed-care patterns and in several individual and work-related characteristics. Moral sensitivity and perceived organizational support showed heterogeneous, profile-specific associations with missed nursing care. These findings suggest that management strategies may benefit from considering differences across nurse subgroups. Given the cross-sectional design, however, the identified profiles should not be interpreted as developmental stages or evidence of transitions between profiles. Longitudinal and interventional studies are needed to confirm temporal patterns and evaluate the effectiveness of profile-informed interventions.

## Supplementary Information

Below is the link to the electronic supplementary material.

Supplementary Material 1

Supplementary Material 2

Supplementary Material 3

Supplementary Material 4

Supplementary Material 5

## Data Availability

The datasets used and analyzed during the current study are available from the corresponding author upon reasonable request.

## Abbreviations

ICU: Intensive Care Unit
MNC: Missed Nursing Care
LPA: Latent Profile Analysis
JD-R: Job Demands-Resources Model
AIC: Akaike Information Criterion
BIC: Bayesian Information Criterion
aBIC: adjusted Bayesian Information Criterion
LMR: Lo-Mendell-Rubin Test
BLRT: Bootstrap Likelihood Ratio Test
OR: Odds Ratio
CI: Confidence Interval
