# Supplementary material for: Latent profiles and influencing factors of missed nursing care among ICU nurses
Source: BMC Nurs. 2026 Aug 28;25:993. doi: 10.1186/s12912-026-05308-1 (PMC13681808; doi:10.1186/s12912-026-05308-1)
Supplement: Supplementary file 1 [file 12912_2026_5308_MOESM1_ESM.pdf]

General Information Questionnaire

Please select the option that best describes your situation by ticking "√" in the corresponding box.

1. Gender:

□ Male □ Female

2. Age (years):

□ ≤25 □ 26–30 □ 31–35 □ 36–40 □ ≥41

3. Marital status:

□ Married □ Unmarried □ Other

4. Education level:

□ Technical secondary school □ Junior college □ Bachelor □ Postgraduate

5. Professional title:

□ Nurse □ Senior nurse □ Nurse-in-charge □ Associate chief nurse

6. Type of ICU:

□ Medical ICU □ Surgical ICU □ General ICU □ Emergency ICU □ Other ICU

7. Years of working experience in ICU:

□ 1–4 years □ 5–9 years □ ≥10 years

8. Average number of patients assigned per shift:

□ 1–2 patients □ 3–4 patients □ ≥5 patients

9. Average monthly overtime hours (working beyond scheduled hours):

□ No overtime □ 1–5 hours □ 6–10 hours □ 11–15 hours □ >15 hours

10. Certified ICU specialist nurse:

□ Yes □ No

11. Satisfaction with departmental human resource allocation:

□ Very satisfied □ Satisfied □ Dissatisfied □ Very dissatisfied

12. Satisfaction with departmental material resource allocation:

□ Very satisfied □ Satisfied □ Dissatisfied □ Very dissatisfied

13. Satisfaction with departmental communication climate:

□ Very satisfied □ Satisfied □ Dissatisfied □ Very dissatisfied
