# Supplementary material for: Latent profiles and influencing factors of missed nursing care among ICU nurses
Source: BMC Nurs. 2026 Aug 28;25:993. doi: 10.1186/s12912-026-05308-1 (PMC13681808; doi:10.1186/s12912-026-05308-1)
Supplement: Supplementary file 2 [file 12912_2026_5308_MOESM2_ESM.docx]

**Supplementary Table1** Mean scores of the top five items of missed nursing care among ICU nurses

| Item No. | Item Content | Item mean score (x̅±s) |
| --- | --- | --- |
| A17 | Psychosocial assessment of critically ill patients | 2.39±1.03 |
| A36 | Assisting and guiding patients to initiate early rehabilitation | 2.38±1.22 |
| A8 | Assessment of patient muscle strength | 2.27±0.91 |
| A25 | Communication with patients and psychological care | 2.17±0.98 |
| A3 | Assessment of patient pain | 2.12±0.96 |
