# Supplementary material for: Latent profiles and influencing factors of missed nursing care among ICU nurses
Source: BMC Nurs. 2026 Aug 28;25:993. doi: 10.1186/s12912-026-05308-1 (PMC13681808; doi:10.1186/s12912-026-05308-1)
Supplement: Supplementary file 3 [file 12912_2026_5308_MOESM3_ESM.docx]

**Supplementary Table 2** Mean scores of the top five perceived reasons for missed nursing care among ICU nurses

| Item No. | Item Content | Item mean score (x̅±s) |
| --- | --- | --- |
| B1 | High workload of nurses | 3.80±0.54 |
| B6 | High frequency of patient condition changes and rescue events | 3.02±0.98 |
| B7 | Excessive patient admission/discharge activities | 2.91±1.00 |
| B24 | Excessive non-nursing work | 2.88±1.05 |
| B11 | Unavailable or inconvenient use of electronic medical records/information systems | 2.80±0.89 |
| B18 | Excessive nursing interruptions | 2.80±1.03 |
