# Supplementary material for: Latent profiles and influencing factors of missed nursing care among ICU nurses
Source: BMC Nurs. 2026 Aug 28;25:993. doi: 10.1186/s12912-026-05308-1 (PMC13681808; doi:10.1186/s12912-026-05308-1)
Supplement: Supplementary file 4 [file 12912_2026_5308_MOESM4_ESM.docx]

**Supplementary Table 3** Top five missed nursing care items among ICU nurses in different latent profiles (N=332, mean ± SD)

| No. | Low MNC Group (n=188) | Moderate MNC Group (n=94) | High MNC Group (n=50) |
| --- | --- | --- | --- |
| 1 | A8 Assessing patient muscle strength  1.90±0.78 | A17 Psychological assessment of critically ill patients  2.79±0.79 | A36 Assisting and guiding patients to initiate early rehabilitation  4.30±0.79 |
| 2 | A17 Psychological assessment of critically ill patients  1.88±0.82 | A36 Assisting and guiding patients to initiate early rehabilitation  2.76±0.92 | A35 Sedation and analgesia management  3.60±0.50 |
| 3 | A3 Assessing patient pain  1.71±0.77 | A8 Assessing patient muscle strength  2.60±0.83 | A17 Psychological assessment of critically ill patients  3.58±0.81 |
| 4 | A25 Communicating with patients and providing psychological care  1.70±0.66 | A6 Assessing patient nutritional status  2.50±0.88 | A25 Communicating with patients and providing psychological care  3.50±0.84 |
| 5 | A36 Assisting and guiding patients to initiate early rehabilitation  1.68±0.71 | A18 Reassessing patients according to disease severity and formulating or updating care plans  2.44±0.73 | A3 Assessing patient pain  3.30±0.86 |
