# Supplementary material for: Latent profiles and influencing factors of missed nursing care among ICU nurses
Source: BMC Nurs. 2026 Aug 28;25:993. doi: 10.1186/s12912-026-05308-1 (PMC13681808; doi:10.1186/s12912-026-05308-1)
Supplement: Supplementary file 5 [file 12912_2026_5308_MOESM5_ESM.docx]

**Supplementary Table 4** Top five perceived causes of missed nursing care among ICU nurses in different latent profiles

| No. | Low MNC Group (n=188) | Moderate MNC Group (n=94) | High MNC Group (n=50) |
| --- | --- | --- | --- |
| 1 | B1 High workload of nurses  3.79±0.55 | B1 High workload of nurses  3.78±0.57 | B1 High workload of nurses  3.86±0.45 |
| 2 | B6 High frequency of disease changes and resuscitation events  2.99±0.99 | B24 Excessive non-nursing tasks  2.95±1.11 | B18 Excessive nursing interruptions  3.30±0.89 |
| 3 | B7 Frequent patient admissions and transfers  2.84±1.00 | B6 High frequency of disease changes and resuscitation events  2.94±1.03 | B6 High frequency of disease changes and resuscitation events  3.26±0.85 |
| 4 | B11 Unavailable or inconvenient electronic medical record/information system  2.84±0.92 | B7 Frequent patient admissions and transfers  2.87±1.06 | B7 Frequent patient admissions and transfers  3.24±0.80 |
| 5 | B24 Excessive non-nursing tasks  2.79±1.06 | B18 Excessive nursing interruptions  2.79±0.99 | B24 Excessive non-nursing tasks  3.10±0.84 |
